# The Perception of Aversiveness of Surgical Procedure Pictures Is Modulated by Personal/Occupational Relevance

**DOI:** 10.1371/journal.pone.0160582

**Published:** 2016-08-12

**Authors:** Juliana Paes, Leticia de Oliveira, Mirtes Garcia Pereira, Isabel David, Gabriela Guerra Leal Souza, Ana Paula Sobral, Walter Machado-Pinheiro, Izabela Mocaiber

**Affiliations:** 1 Laboratory of Cognitive Psychophysiology, Department of Natural Sciences, Institute of Humanities and Health, Federal Fluminense University, Rio das Ostras, RJ, Brazil; 2 Laboratory of Neurophysiology of Behavior, Department of Physiology and Pharmacology, Biomedical Institute, Federal Fluminense University, Niterói, RJ, Brazil; 3 Laboratory of Psychophysiology, Department of Biological Sciences, Federal University of Ouro Preto, Ouro Preto, MG, Brazil; 4 Department of Engineering, Institute of Science and Technology, Federal Fluminense University, Rio das Ostras, RJ, Brazil; University of Florida, UNITED STATES

## Abstract

It is well established that emotions are organized around two motivational systems: the defensive and the appetitive. Individual differences are relevant factors in emotional reactions, making them more flexible and less stereotyped. There is evidence that health professionals have lower emotional reactivity when viewing scenes of situations involving pain. The objective of this study was to investigate whether the rating of pictures of surgical procedure depends on their personal/occupational relevance. Fifty-two female Nursing (health discipline) and forty-eight Social Work (social science discipline) students participated in the experiment, which consisted of the presentation of 105 images of different categories (e.g., neutral, food), including 25 images of surgical procedure. Volunteers judged each picture according to its valence (pleasantness) and arousal using the Self-Assessment Manikin scale (dimensional approach). Additionally, the participants chose the word that best described what they felt while viewing each image (discrete emotion perspective). The average valence score for surgical procedure pictures for the Nursing group (M = 4.57; SD = 1.02) was higher than the score for the Social Work group (M = 3.31; SD = 1.05), indicating that Nursing students classified those images as less unpleasant than the Social Work students did. Additionally, the majority of Nursing students (65.4%) chose “neutral” as the word that best described what they felt while viewing the pictures. In the Social Work group, disgust (54.2%) was the emotion that was most frequently chosen. The evaluation of emotional stimuli differed according to the groups' personal/occupational relevance: Nursing students judged pictures of surgical procedure as less unpleasant than the Social Work students did, possibly reflecting an emotional regulation skill or some type of habituation that is critically relevant to their future professional work.

## Introduction

It is believed that emotions are organized around two motivational systems, one appetitive and one defensive, which allows individuals to adequately interact with ongoing circumstances that either promote or threaten survival [1]. The defensive system is activated in contexts involving threat, whereas the appetitive system is activated in contexts that promote survival (e.g., sustenance, procreation and nurturance). The motivational model assumes two dimensions: (a) hedonic valence (i.e., pleasant–appetitive motivation or unpleasant–defensive motivation) and (b) arousal (i.e., degree of motivational activation). Thus, evaluations of pleasure or displeasure in relation to a certain stimulus indicate which motivational system is predominant, and judgments of arousal point to the intensity of motivational activation [2].

A vast number of papers on the psychophysiology of emotion have shown that affective pictures drive the activity of brain networks and impact behavior [3, 4, 5, 6]. Lang et al. developed a catalogue with hundreds of emotional and neutral pictures, the International Affective Picture System—IAPS (Center for the Study of Emotion and Attention [CSEA]) [7], and developed a scale, the Self-Assessment Manikin (SAM), to evaluate the pleasure (hedonic valence) and the emotional arousal evoked when viewing these pictures [8]. The SAM has been used extensively to measure subjective responses to distinct emotional patterns evoked by pictures. The specific picture content represents unpleasant pictures (e.g., pollution, loss, illness), neutral pictures (people and objects) and pleasant pictures (e.g., nature, sports, food). The IAPS has been used in a variety of studies, including those involving clinical samples and studies probing the effects of distinct physiological conditions (e.g., sleep deprivation) on subjective ratings of emotional pictures.

In this vein, several studies have employed picture viewing and subjective rating to induce and assess emotion, respectively, in volunteers. In addition to the existence of the two motivational systems already mentioned for organizing behavior, *personal relevance* is a critical factor that determines affective responses [9]. Studies from our group have shown that positive affect traits [10, 6], anxiety traits [11], trauma experience [12, 13], severity of post traumatic symptoms [14], resting vagal tone and resilience traits [15] are able to modulate emotional response in several experimental paradigms. These findings are in line with several studies that show the critical role of individual differences in affective response and regulation [16, 17, 18 19]. For instance, Drabant et al. [16] showed that the greater use of reappraisal in everyday life was related to decreased amygdala activity and increased prefrontal and parietal activity during the processing of negative emotional facial expressions, which might predict successful coping with emotional challenges.

Emotional response is critically influenced by *contextual* factors. Rather than being stereotyped, emotions are flexible and modulated by circumstances [9]. It has been shown that the psychophysiological impact (measured by heart rate, brain activation and evoked potential indexes) of highly unpleasant scenes (mutilation pictures) can be attenuated when previous information describes them as fictitious. This prior information allows participants to effortlessly adjust their affective response in the current context [10, 20, 21]. When participants receive this “safety cue”, they are extrinsically induced to reevaluate and down-regulate the impact or relevance of these stimuli. Safety cues can indicate the absence of threat [22] and are believed to provide information about potential positive outcomes [23]. Thus, even pictures of mutilated bodies that represent an imminent threat and are known to engage strong defensive reactions [2] are susceptible to contextual regulation.

A recent study provided additional evidence that empathy to pain can be affected by emotional regulation mechanisms [24]. The authors hypothesized that healthcare professionals would respond differently to pain scenes, possibly reflecting emotion regulation skills. Event-related potentials (ERP) were collected from physicians and a non-physician matched group while the participants viewed visual stimuli depicting body parts being pricked by a needle (pain) or touched by a cotton swab (no pain). The results showed early P3 differentiation between the pain and no-pain conditions over centro-parietal regions in the control participants, supporting the idea that empathy to pain modulates an ERP component that is classically associated with attentional processing. In contrast, no such ERP-differentiated responses were detected in the physician group. It was proposed that physicians' down-regulation of the emotional response reduced negative arousal related to the pain of others and that this ability has beneficial consequences for health assistance. In fact, for healthcare professionals who lack emotion regulation abilities, repeated exposure to the suffering of others may be related to the adverse consequences of personal distress and burnout syndrome.

Finally, the role of empathy in health professionals and graduation students has also received considerable attention [25, 26]. Health care professionals must constantly face the challenge of finding a balance that allows them to identify and recognize the details of a patient’s pain experience and resonate with it without becoming emotionally overinvolved in a way that could prevent effective medical treatment and even lead to burnout [27]. We assessed empathy traits to investigate their modulation in affective ratings to surgical procedure pictures, which are relevant to the future occupation of Nursing students. In academic/professional settings, unaffected concern for the patient is encouraged [28] based on the assumption that oversensitive observers of others’ pain may face difficulties in delivering effective helping behavior and may respond with high distress [29].

In the present work, we used the SAM [8], a standardized subjective rating protocol, to investigate the emotional response to a set of pictures whose content is relevant to health discipline students, namely, *surgical procedure pictures*. These pictures depict parts of bodies with blood, body envelope violation, body perforation, puncture and the presence of secretions in the context of a surgical center in the presence of lancets, scalpels and surgical gloves. Although the pictures contain deep cuts, blood, inner tissue, and incisions, they assume a positive outcome (safety cue) because the images are in the context of a surgical center operated by health professionals.

In addition to the classical dimensional approach, we consider the concept that images can elicit specific patterns of somatic (e.g., facial muscle change) and autonomic activation associated with specific emotions [30]. Additionally, it is conceivable that the dimensional and state-specific emotion views are not mutually exclusive but are hierarchically related [31]. Thus, after rating the image in terms of pleasure and arousal, the participant picked one of seven specific emotions that best described the feeling evoked by each picture.

In summary, we expected that picture assessment would be flexible and modulated by personal/occupational relevance. To probe this proposal, we compared the pictures' ratings between health discipline students (Nursing) and a matched group of social science discipline students (Social Work). Our hypothesis was that the two groups would differently rate the images as a function of their relevance and occupational scope.

## Methods

### Participants

Fifty-two female Nursing students with a mean age of 21.44 years (SD = 3.71) and forty-eight female Social Work students with a mean age of 28.69 years (SD = 10.64) took part in the experiment. Volunteers were selected from students from the Fluminense Federal University, Brazil. They had normal or corrected-to-normal vision, reported no psychiatric or neurological problems and were not taking medication that acted on the nervous system. For the empathy trait analysis, 3 participants (Social Work group) were excluded due to not having completed the IRI scale. Participants were naïve with regard to the purpose of the experiment. They were told only that the experiment consisted of the evaluation of emotional pictures. All participants provided written consent, by means of an approved consent form to take part in the study. Ethical approval for the study was obtained from the Ethics Committee of the School of Medicine/Antônio Pedro University Hospital, Fluminense Federal University.

### Apparatus

The experiment was conducted in a dimly lit room with comfortable desks placed in rows in front of a slide projection screen. The desks were arranged in such a manner that the screen was completely visible to every participant. The distance between the projection and the first line of desks was 3.00 m, and the distance between the projection and the last line of desks was 7.20 m. A microcomputer containing the software E-prime 2.0 (Psychological Software Tools, Inc.) controlled both the order and the timing of the stimuli presentation. Using an Epson projector, the pictures were displayed on a white screen so that the stimuli had an average size of 2.0 m (horizontal) and 1.5 m (vertical).

### Stimuli

#### Surgical procedure pictures

We used 25 pictures of surgical procedure acquired at *thinkstock*^®^ (http://www.thinkstockphotos.com) by Getty Images. These pictures had specific characteristics: body envelope violation; the presence of body/human body parts; blood or secretion; and the presence of surgical center objects (gloves, scalpel and pincers).

#### IAPS pictures

The surgical procedure pictures were interspersed with pictures with different emotional categories taken from the International Affective Picture System (IAPS) [7]. Eighty pictures were selected that were balanced in the degree of both pleasantness (hedonic valence) and arousal. The selected pictures comprised distinct content, including content typically rated as pleasant (nature, family, food, sports, adventure, attractive men and attractive women), neutral (people and objects) and unpleasant (pollution, illness, loss, accidents, contamination, attacking animals, attacking humans, mutilated bodies). The final set of pictures comprised 32 pleasant images (varying from low to high arousal), 32 unpleasant pictures (from low to high arousal), 16 neutral pictures, and the 25 surgical procedure pictures already mentioned for a total of 105 pictures. The physical properties of the pictures did not differ with regard to brightness [F _(3,101)_ = 0.53, p = 0.66]. M = 100.59, SD = 44.53 for positive images; M = 90.31, SD = 29.43 for negative images; M = 93.88, SD = 33.82 for neutral images and M = 99.00, SD = 31.28 for surgical procedure images. For the contrast, we observed a main effect of category [F _(3,101)_ = 2.91, p < 0.05]. When conducting the specific comparisons in the post hoc analysis (Newman Keuls), the following probability values were obtained: surgical procedures M = 13.00, SD = 5.52 *vs*. positive M = 17.11, SD = 9.20, p = 0.07; *vs*. negative M = 12.49, SD = 6.69, p = 0.93; *vs*. neutral M = 18.02, SD = 9.09, p = 0.07.

### Instruments

#### Self-assessment manikin (SAM; [8])

The images were evaluated using the paper-and-pencil version of the SAM, which consists of pictorial drawings of manikins representing the dimensions of hedonic valence and emotional arousal. For each dimension, there is a row of five figures interleaved by blank spaces, yielding nine intensity levels. For the hedonic valence dimension, the manikins exhibit expressions that range from “smiling-happy” (score = 9) to “frowning-unhappy” (score = 1). For the emotional arousal dimension, the expressions of the manikins range from an “excited wide-eyed” figure (score = 9) to a “relaxed-sleepy” figure (score = 1). Multiple sessions with small groups of different participants (ranging in size from 20 to 30 per session) were performed to obtain the rates related to the pictures of interest.

#### Trait empathy

The participants’ trait empathy was measured with the Portuguese version of the Interpersonal Reactivity Index (IRI) [32]. The IRI consists of 28 items rated on a 5-point scale (does not describe me well—describes me very well). The items are arranged into four subscales with seven items. Each subscale measures a distinct component of empathy: empathic concern, personal distress, perspective taking and fantasy scale.

### Procedure

The experimental design consisted of the visualization and assessment of images of different categories. Upon their arrival, the participants were asked to be seated and to read and sign the consent form if they were willing to participate in the study. Each participant then received a copy of the instructions and a rating booklet. The participants read the instructions silently. The investigators who conducted the sessions were trained to provide similar explanations about the experiment. Before the beginning of the experiment, participants read written instructions that they would see images of distinct contents. Then, a didactic video explained the incoming task was shown in which all rating possibilities in the pleasure and arousal dimensions were mentioned. A training was subsequently performed using 3 pictures from the IAPS that aimed exclusively to allow the participants to appropriately use the SAM scale after viewing each image. Thus, it had the main objective of permitting the participant to test and control the viewing and rating time. The pictures were randomly selected from a set of eight pictures (5830, 1500, 1510, 1463, 1600, 1560, 1340, 5811).The experiment began, and the participants rated a total of 105 images. Of these images, 25 belonged to the category of interest: *surgical procedure*. The other 80 pictures were obtained from IAPS and were used as a background to provide participants with a rough range of the types of content and to anchor the emotional rating scales. Participants were asked to rate each picture along the dimensions of hedonic valence and emotional arousal using the paper-and-pencil version of the SAM scales [8] (dimensional approach). Additionally, specific (discrete) emotion was assessed by asking the participants to choose from 7 words the one that best described what they felt while viewing each image: anger, disgust, fear, happiness, sadness, surprise and neutral. The words were based on lists that consider emotions to be discrete states that differ in terms of appraisal, facial expressions and action tendencies (e.g., [33, 34]).

Each rating trial began with the preparation slide “Rate picture number 1”, which lasted for 3 seconds and was followed by a 6 s picture viewing period. During the next 15 s, participants were asked to rate each picture in the dimensions of hedonic valence, emotional arousal and specific (discrete) emotion.

The sequential order of the picture presentation was pseudo-randomized with the constraint that specific content could not be repeated more than twice consecutively. The completion of each experimental session lasted approximately 1 hour.

## Data Analyses

First, the average ratings from the participants were computed for each picture, representing the mean degree of valence and arousal attributed to each picture. Spearman correlations between the average rating of IAPS pictures obtained in the present sample and those reported for North Americans were conducted to assess the consistency of an image’s distribution in the bi-dimensional model proposed by Greenwald et al. [35]. According to this model, the ratings of valence and arousal of a heterogeneous emotion-laden group of pictures plotted in a Cartesian plan are disposed in vectors that point in two directions, representing a "boomerang" shape. The upper arm of the boomerang indexes appetitive (approach-like) motivation, and the lower arm indexes defensive (avoidance-like) motivation.

We compared between-group difference in arousal and valence ratings using a repeated-measures ANOVA with a between-factor (Nursing vs. Social Work students) and a within-factor (surgical, positive, negative or neutral pictures). The Greenhouse- Geisser correction for nonsphericity was applied. Partial eta-squared (η^2^) was calculated as an estimate of effect size, and, when appropriate, the Newman-Keuls post-hoc test was performed. Chi-square tests were employed to examine associations between group and specific emotion choices among the 7 possible words.

We compared the mean scores of each IRI subscale between the two groups by means of independent t-tests. Spearman correlations were conducted between pleasure and arousal ratings and empathy trait scores.

Statistical significance for all tests was evaluated at the α = .05 level.

## Results

**Fig 1:**
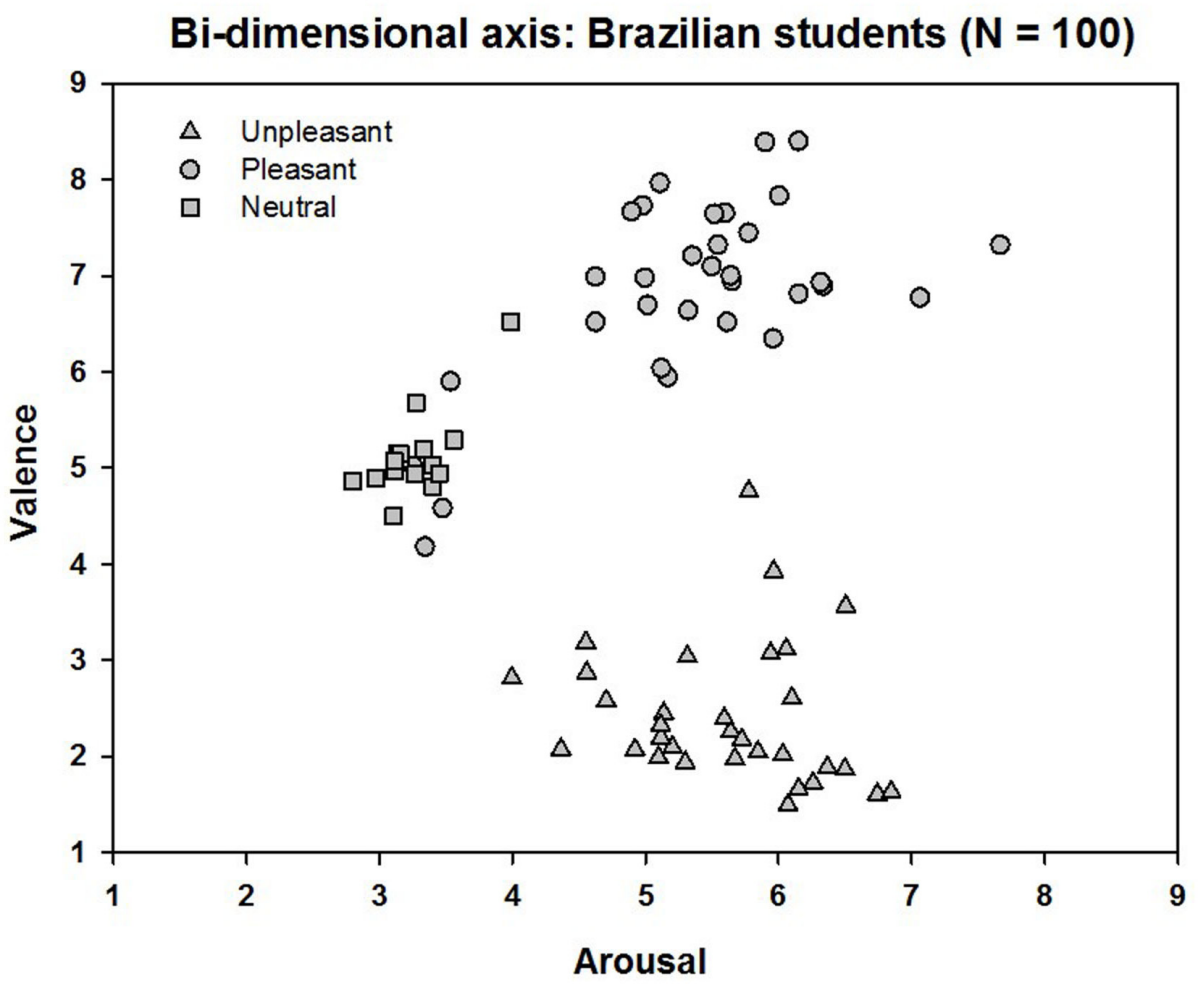
Affective space: bi-dimensional plot of each image as a function of its mean hedonic valence (y axis) and arousal ratings (x axis). Each point in the graph represents the mean rating for each picture given by all students (N = 100). Participants judged 80 IAPS images. IAPS unpleasant pictures (triangles); pleasant pictures (circles); neutral pictures (squares).

The average score for each IAPS picture was compared with that reported for North Americans. The correlation was high for both the hedonic valence (rho = 0.97, p < 0.001) and the emotional arousal (rho = 0.83, p < 0.001) dimensions. A plot of the mean scores for valence and arousal on a Cartesian plane (Fig 1) revealed the same “boomerang”-shaped distribution described by Lang et al. [7], who proposed that the upper arm of the boomerang indexes appetitive (approach-like) motivation and the lower arm indexes defensive (avoidance-like) motivation. Thus, stimuli judged as pleasant ranged in the arousal scale from relatively calm to highly arousing, and unpleasant stimuli ranged from calm to highly arousing.

The repeated-measures ANOVA for valence revealed a main effect of group [F_(1,98)_ = 15.98, p < 0.001, η^2^ = 0.14] and category [F_(3,294)_ = 621.88, p < 0.0001, ε = 0.71, η^2^ = 0.86]. Additionally, there was a statistically significant interaction between group and category [F_(3,294)_ = 16.80, p < 0.0001, ε = 0.71, η^2^ = 0.15]. The Newman Keuls analysis showed that Social Work students judged surgical procedure images as more unpleasant than Nursing students did (M = 3.31 SD = 1.05 and M = 4.57 SD = 1.02, respectively; p < 0.001). In fact, the mean valence obtained by Nursing students for the surgical images corresponded to "neutral" pictures, whereas the average score obtained by the Social Work group corresponded to "negative" images.

**Fig 2:**
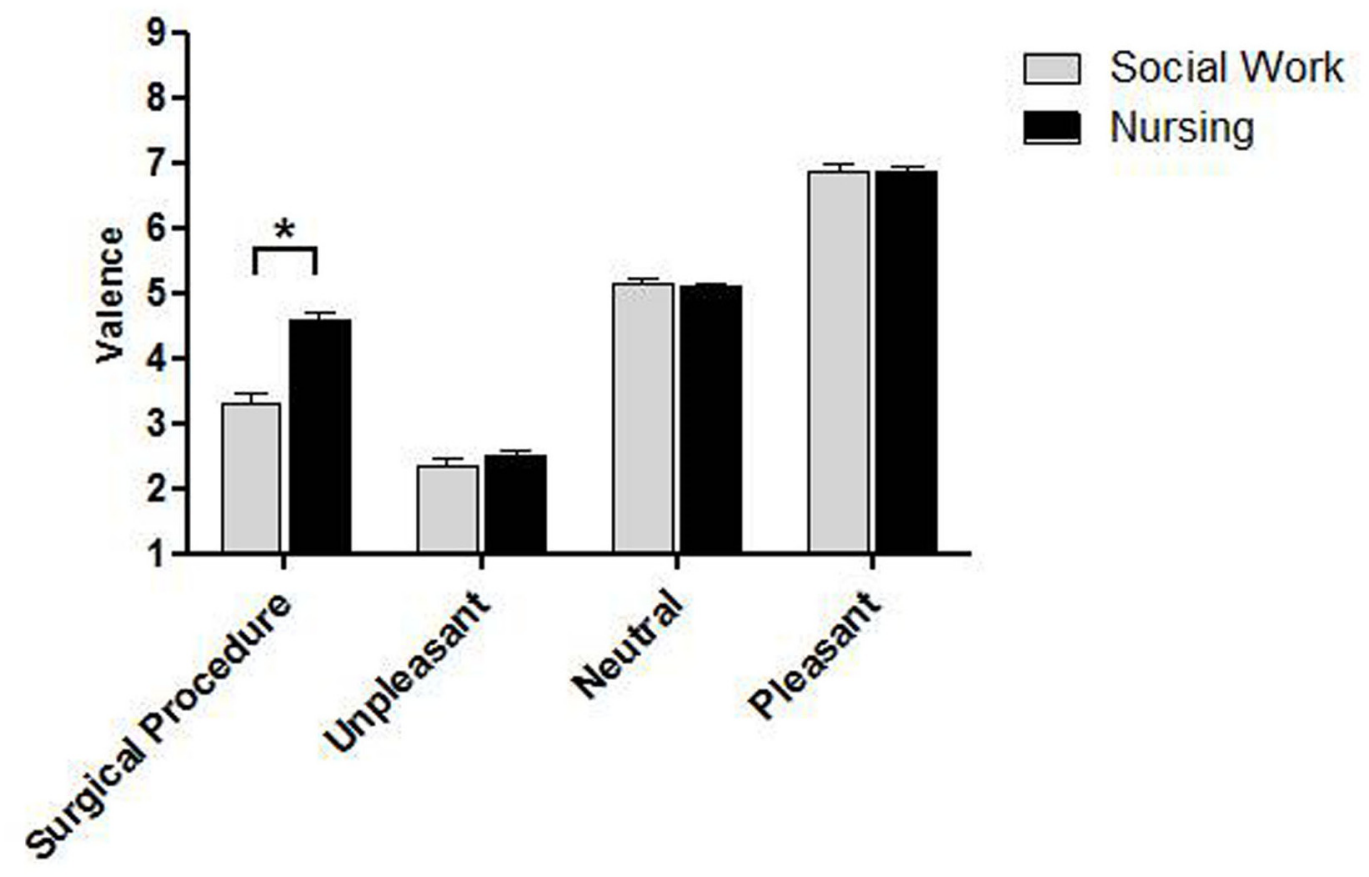
Valence rating for the four categories. The asterisk represents statistically significant differences (p < 0.05), and the bars indicate the standard error of the mean.

The rating of positive (M = 6.85 SD = 0.84 and M = 6.84 SD = 0.60, for Social Work and Nursing students, respectively; p = 0.96), negative (M = 2.34 SD = 0.73 and M = 2.49 SD = 0.73, respectively; p = 0.36) and neutral (M = 5.13 SD = 0.62 and M = 5.10 SD = 0.40, respectively; p = 0.87) images was not modulated by the group factor (Fig 2).

The repeated-measures ANOVA for arousal did not reveal a main effect of group [F_(1,98)_ = 0,03, p < 0.863, η^2^ = 0.0003]. There was a main effect of category [F_(3,294)_ = 81.03, p < 0.0001, ε = 0.87, η^2^ = 0.45]: surgical procedure images were more arousing than neutral images (M = 4.82 SD = 1.54 and M = 3.30 SD = 1.54, respectively; p < 0.0001), but less arousing than positive (M = 5.35 SD = 1.52, p < 0.01) and negative images (M = 5.63 SD = 1.51, p < 0.0001). Positive and negative images did not differ in terms of arousal ratings (p = 0.1).There was no statistically significant interaction between group and category [F_(3,294)_ = 1.79, p = 0.86, ε = 0.87, η^2^ = 0.02]. The rating of positive (M = 5.21 SD = 1.63 and M = 5.50 SD = 1.39, for Social Work and Nursing students, respectively), negative (M = 5.87 SD = 1.55 and M = 5.41 SD = 1.44, respectively), neutral (M = 3.31 SD = 1.37 and M = 3.30 SD = 1.68, respectively) and surgical procedure (M = 4.82 SD = 1.38 and M = 4.83 SD = 1.68, respectively) images was not modulated by the group factor.

**Fig 3:**
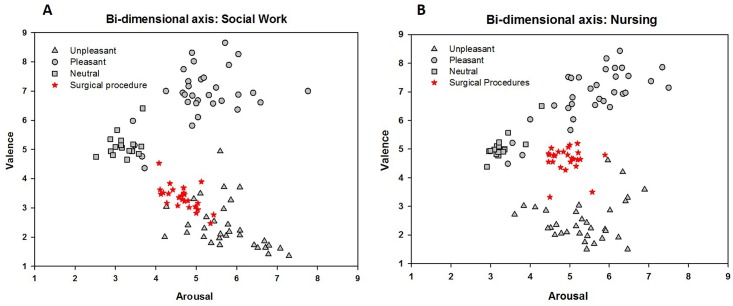
Affective space: Bi-dimensional space consisting of hedonic valence (y axis) and arousal ratings (x axis) for each picture. For the Social Work group (A), the majority of surgical procedure pictures occupy the lower "defensive motivational system" arm of the space, whereas for the Nursing students (B), they are closer to a neutral "less emotional" area. Each point in the graph represents the mean rating for each picture. IAPS unpleasant pictures (triangles); pleasant pictures (circles); neutral pictures (squares); surgical procedure pictures (stars). (A) Social Work; (B) Nursing.

The plot of the mean scores for valence and arousal ratings on the Cartesian plane suggests that the distribution of surgical procedure pictures differs in the bi-dimensional approach of emotion [36] as a function of the group. As shown in Fig 3, surgical procedure pictures occupy distinct positions in the bi-dimensional space in each group. For the Social Work group, they occupy the lower "defensive" arm of the bi-dimensional space (Fig 3A), whereas for the Nursing students, they occupy a central "less emotional" area (Fig 3B).

**Fig 4:**
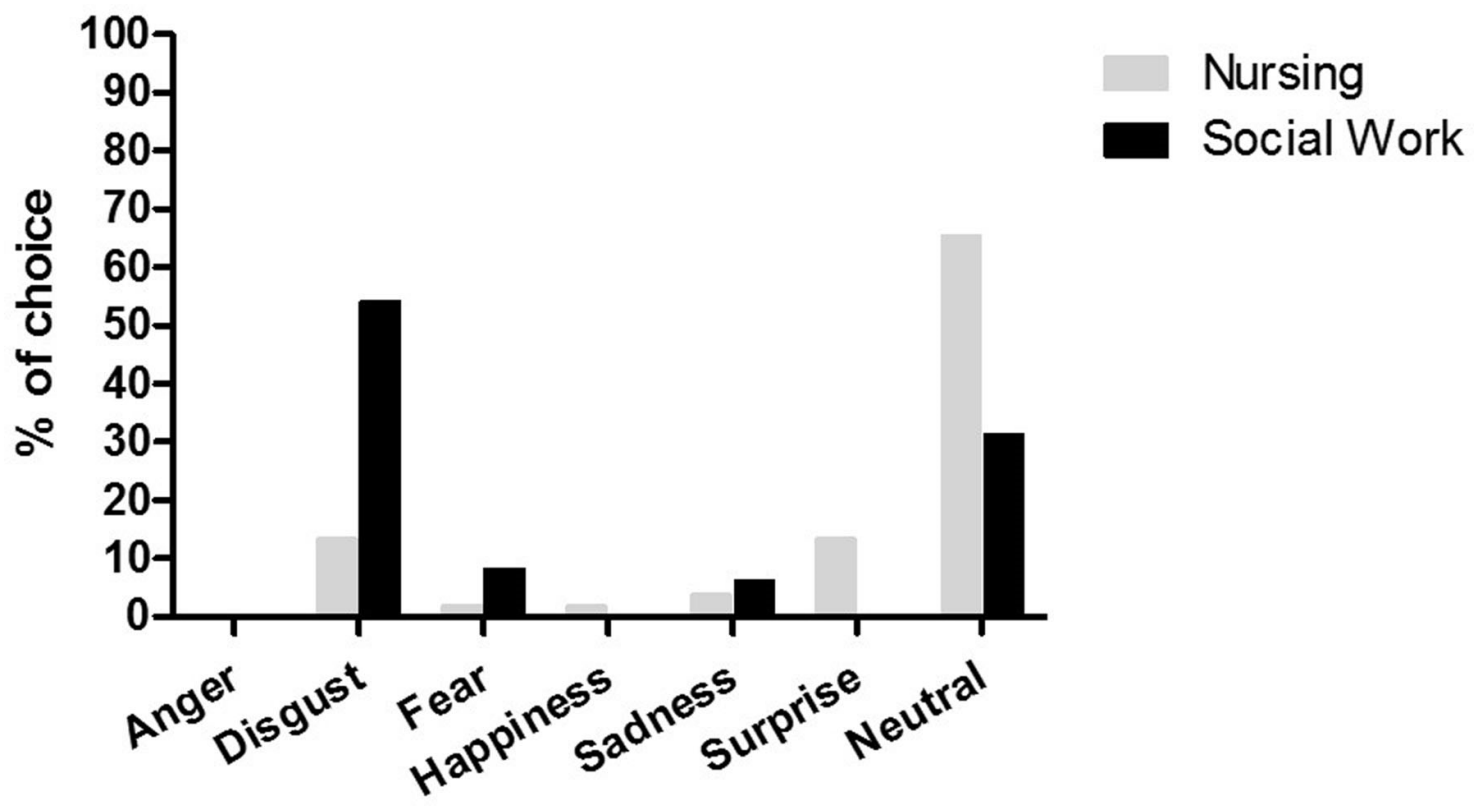
Frequency distribution choice of discrete emotions for the Nursing and Social Work groups: neutral (65.4% and 31.25%, respectively); surprise (13.46% and 0%, respectively); sadness (3.84% and 6.25%, respectively); happiness (1.92% and 0%, respectively); fear (1.92% and 8.33%, respectively); disgust (13.46% and 54.17%, respectively). None of the groups chose anger as a discrete emotion.

The chi-square test showed that distinct categorical choices (specific emotion) were associated with each group of students: χ2 = 28.19, df = 5, p < 0.001. As shown in Fig 4, most of the Nursing students assessed surgical procedure pictures as neutral (65.4% of the students). In contrast, the majority of Social Work students classified these pictures as disgusting (54.2%).

This findings fit well with the dimensional approach results, in which Nursing students evaluated surgical procedure pictures mostly as less emotional and Social Work students evaluated them as more unpleasant. Here, the Social Work group was also associated with the consistent choice of the word “disgust”, possibly explaining the finding that the pictures were rated by them as more unpleasant in the dimensional approach. A significant difference in empathy levels between Social Work (M = 17.09 SD = 6.35) and Nursing (M = 14.58 SD = 4.30) students was found for the personal distress subscale only (t = 2.30, p < 0.03). No differences were observed for the fantasy scale (M = 16.91 SD = 5.30 and M = 18.35 SD = 6.25, p = 0.23), empathic concern (M = 21.80 SD = 3.97 and M = 21.48 SD = 4.58, p = 0.72), and perspective taking (M = 16.95 SD = 4.97 and M = 16.77 SD = 4.44, p = 0.85).

Because the personal distress (PD) subscale scores were significantly different between the two groups, we conducted a correlation analysis between the PD score and valence and arousal ratings for each category (neutral, negative, positive and surgical procedure). For the valence dimension (Nursing group), we observed a significant correlation between valence and PD score in the surgical procedure category only (rho = −0.35, p = 0.02). No significant correlations were observed between valence and PD score for the neutral (rho = −0.18, p = 0.19), negative (rho = 0.12, p = 0.40), and positive (rho = −0.02, p = 0.89) categories. For the arousal dimension (Nursing group), no significant correlations were observed for the surgical procedure (rho = 0.06, p = 0.62), neutral (rho = 0.02, p = 0.88), negative (rho = 0.24, p = 0.09), and positive (rho = −0.03, p = 0.82) categories.

For the valence dimension (Social Work group), no significant correlations were observed between pleasure and PD score for the surgical procedure (rho = −0.25, p = 0.10), neutral (rho = 0.14, p = 0.34), negative (rho = -0.15, p = 0.32), and positive (rho = 0.16, p = 0.30) categories. For the arousal dimension (Social Work group), no significant correlations were observed between arousal and PD score for the surgical procedure (rho = 0.28, p = 0.06), neutral (rho = 0.02, p = 0.87), negative (rho = 0.10, p = 0.53), and positive (rho = 0.17, p = 0.26) categories.

## Discussion

In the present work, we used a well-established methodology in emotion studies to assess the evaluation of surgical procedure pictures by two groups of undergraduate students. As expected, the participants’ judgments of IAPS images were highly correlated to the North American group ratings [7], guaranteeing the appropriateness of the methodology. The classical boomerang graphic was obtained, with pictures of all contents used as a background, occupying both arms of each “motivational system”. Interestingly, according to the bidimensional organization of emotion, surgical procedure pictures were judged as *less emotional (less unpleasant)* by Nursing students compared to Social Work students. Both groups similarly judged the images as *moderately arousing*. The rating of the pleasantness of the other categories of images (neutral, negative and positive) was not modulated by group, indicating the specificity of the surgical procedure images, which differed in terms of their personal/occupational relevance. Additionally, according to the categorical (discrete emotions) approach, the word *neutral* was the most frequently chosen word by Nursing students to best describe what they felt while viewing pictures of surgical procedure, in accordance with the findings obtained in the bi-dimensional approach. The Social Work group, in contrast, was mainly associated with the choice of the word *disgust*. This agreement is interesting from the perspective that both approaches (dimensional and state-specific emotion) are not mutually exclusive but are hierarchically related [31]. The finding that surgical procedure pictures were judged as *less unpleasant* is in accordance with our assumptions that Nursing students would exhibit a differentiated response pattern. A possible explanation for the results could be an emotional regulation mechanism when viewing surgical procedure pictures, due to the occupational relevance of the pictures to Nursing students. This possible mechanism is supported by the results obtained by Decety et al. [24], who showed that physicians, compared to control participants, did not exhibit the emotional modulation of an ERP visual component (P300) while viewing pain scenes. The authors interpreted this finding as an evidence of the emotional regulation necessary in physicians to maintain health care assistance without becoming mentally and emotionally exhausted. In fact, compassion fatigue (or secondary traumatic stress) has been referred as a natural consequence of caring for clients who are in pain [36]. However, it has also been said that caring and compassion might exert a cost on practitioners, 'reducing their capacity or interest in bearing the suffering of others' [37]. This reduction may be achieved through distinct emotional mechanisms. The emotional regulation mechanism is a likely interpretation but it is not the only explanation for the obtained findings. We cannot rule out the occurrence of a habituation of emotional reactions towards aversive contents as a result of being routinely exposed to that content.

The Social Work group, in contrast, rated the surgical procedure pictures as *unpleasant*. We believe that although the pictures had some safety cues, the aversiveness imposed by the perforation, body envelope violation and blood were stronger influences on picture judgment to this group. These elements are likely to have a greater impact on Social Work students because they are not trained to manage them. As clearly observed in the two-dimensional affective space, surgical procedure images were situated in the lower arm of the boomerang, which means that they activated the defensive motivation [2, 38]. In contrast, for the Nursing students, the images were situated in the central part of the boomerang, close to the position occupied by neutral pictures. This difference emphasizes the fact that occupational context and personal relevance modulate the perception of pleasantness. The finding is noteworthy because surgical procedure pictures represent a common situation in a Nursing career, in which health professionals must overcome their own discomfort to provide assistance to the patient, which requires some type of emotional regulation mechanism.

Additionally, the relative “neutrality” of the judgement of the surgical procedure images by Nursing students must be viewed with caution because it has been proposed that some pictures judged as neutral according to pleasantness dimension are, in fact, ambiguous stimuli [39]. This ambiguity is also referred to as ambivalence and suggests that people may experience instances in which they feel both positive and negative. Thus, a neutral rating along a bipolar scale may reflect the presence of both negativity and positivity (ambivalence). The surgical procedure images could have been evaluated by Nursing students as both positive (in association with the desired aspects of their occupation) and negative (in association with the aversive elements of the pictures themselves, such as blood and perforation), producing a less “relatively neutral” score.

The present study showed no group effect in the arousal ratings. There was a main effect of category, in which surgical procedure images were rated as more arousing than neutral IAPS pictures, but slightly less arousing than emotional (pleasant and unpleasant) images. Thus, despite being judged as *less unpleasant* by Nursing students, the images cannot be considered unemotional/neutral because they were moderately arousing to both groups. Although the Nursing participants labeled the images as “neutral” in the categorical approach and judged them as *less unpleasant* in the bidimensional view, they were still aroused/activated by the images. According to the motivation systems approach, the arousal level indexes the degree of approach/avoidance predisposition [2]. Regarding Nursing students (future nurses) one can speculate that it is important to achieve some level of activation to ensure action toward this type of situation (i.e., helping ill people). We believe that nurses must have to overcome automatic avoidance tendencies toward surgical and wound materials, which presumably activate the defensive system. As a function of their occupation and possibly prosocial predispositions, nurses must have an approach attitude to ensure health assistance [40; 41]. Thus, despite having rated the pictures as *less unpleasant*, Nursing students continued to judge them as moderately arousing, as Social Work students did.

In addition to the dimensional approach, the results from discrete emotion choices also provided evidence that Nursing students exhibited a different pattern of evaluation while viewing surgical procedure pictures. The word “neutral” was the most frequently chosen by Nursing students to describe what they felt while viewing pictures of surgical procedure, paralleling the SAM ratings. The second most frequently chosen discrete emotion was “surprise”, possibly reflecting the interest experienced by the subjects while viewing a scene associated with their career. The Social Work group, in contrast, chose “disgust” as the most common emotion to describe what they felt while viewing the pictures. Disgust is a basic emotion associated with an aversive state evoked by repulsive stimuli [42] that protects the physical bodies of organisms from sickness and death and thereby promotes survival [43]. Facial expressions of blood phobics while viewing surgical videos have been more closely associated with disgust than with fear [44]. Tolin et al. [45] showed that blood phobics rated injection pictures as more fearful and disgusting than other groups did, with disgust rated as the dominant emotion. These findings from previous studies are in agreement with our self-reported results in which we showed that differences in ratings can be observed as a function of the personal/occupational of the images.

The surgical procedure images employed in the present study contained blood, body envelope violations and/or secretions, similar to mutilated pictures; that is, they included elements that normally elicit disgust. However, these pictures presented bodies or parts of bodies being manipulated in the context of health assistance. Thus, although the pictures contained elements that would presuppose an evaluation of typically unpleasant images, the group that judged them (Nursing vs. Social Work) was a critical factor in determining their different classifications.

This finding is noteworthy in the case of Nursing students and other healthcare professionals who will face this type of situation in professional settings. Professionals who are strongly over-activated by these types of situations can show delays or even avoidance to these scenes, depriving patients of adequate assistance. Thus, some type of emotional regulation is necessary to this group to overcome defensive/avoidance tendencies and to allow adequate approach behaviors. In fact, we cannot rule out the possibility that some type of *habituation* mechanism critically develops over time in both students and professionals due to daily exposure to situations involving wounds, blood and perforations. Thus, subjective, behavioral and physiological responses to this material can be reduced with chronic exposure which is characteristic of the evolutionary plasticity mechanism known as habituation [45]. This “affective” habituation can function to promote behavior flexibility and prevent maladaptive reactions [46].

We also found that Social Work students scored higher than Nursing students in the personal distress (PD) subscale of the IRI, which measures distinct aspects of empathy. This finding was expected because this subscale contains statements that rely on situations usually confronted by Nursing students (e.g., “Being in a tense emotional situation scares me”). The PD subscale measures the extent to which an individual feels unease as a result of witnessing another person’s emotional distress. In fact, because Nursing students are encouraged to react with dispassionate concern in this type of situation, we expected that they would have a lower PD score. Additionally, we found a moderate inverse correlation between valence ratings and PD scores for the surgical procedure pictures in the Nursing group. This finding indicates that these pictures were rated as more unpleasant when PD scores were higher. The finding that empathy levels (indexed by PD scores) led to an evaluation of the pictures as more aversive is in accordance with the findings that show that high levels of empathy to pain is associated with stronger emotional reactions [47]. This finding fits with the proposal that empathy levels can affect the availability to health assistance. Observers with *average* empathic accuracy scores might be the most effective care deliverers while remaining emotionally adjusted [48] whereas those with high empathy levels may become oversensitive and compromise assistance.

There are some limitations to the present study. First, we performed some adaptations to the procedures recommended by IAPS Tech Manual 2008 [49]. Specifically, during the training, the participants did not rate an example of each valence as recommended by IAPS manual. The training was performed to practice the rating procedure only and it had the main objective of permitting the participants to test and control the viewing and rating time. Furthermore, we displayed 105 images in the rating section to maximize the number of participants for each picture; this was more pictures per section than recommended by the IAPS Tech Manual 2008 [49]. However, we do not believe that these modifications significantly drive our results because we observed high correlations between our owns and the North American subjects’ ratings, and the classical boomerang graphic was obtained. In addition, all participants viewed a standardized video that was designed to avoid any possible bias induced by the experimenter when giving instructions about the rating session. In this video all rating possibilities were presented in the pleasure and arousal dimensions. Finally, is relatively common in the literature to modify the recommendations given by the IAPS manual (for instance, [50, 51, 52]).

Second, we mentioned the emotion regulation mechanism as a possible mechanism to explain our data. However, we did not use emotion regulation questionnaires, rendering this interpretation merely probable.

In summary, we conclude that Nursing students differed in their evaluation of emotional stimuli (surgical procedure images) according to the personal/occupational relevance of the stimuli. They judged these images as less unpleasant when compared to Social Work students and chose the word “neutral” to label the pictures, whereas the Social Work group chose the word “disgust”. Additionally, Nursing students had a lower personal distress trait; the PD levels were associated with an evaluation of the surgical pictures as more aversive. These findings highlight the importance of using meaningful stimuli for the sample studied, and they provide evidence of the flexibility of emotional responses as a function of personal relevance and individual traits.

## Supporting Information

S1 File Ratings for the Nursing and Social Work groups. Valence and Arousal ratings, Specific emotion and Trait of Empathy for both groups (Nursing and Social Work).
(XLS)
